# The impact of fast food marketing on brand preferences and fast food intake of youth aged 10–17 across six countries

**DOI:** 10.1186/s12889-023-16158-w

**Published:** 2023-07-27

**Authors:** Mariangela Bagnato, Marie-Hélène Roy-Gagnon, Lana Vanderlee, Christine White, David Hammond, Monique Potvin Kent

**Affiliations:** 1 School of Epidemiology and Public Health, University of Ottawa, Ottawa, Canada; 2 École de Nutrition, Centre Nutrition, Santé Et Société (Centre NUTRISS), and Institut Sur La Nutrition Et Les Aliments Fonctionnels (INAF), Université Laval, Quebec City, Canada; 3 School of Public Health Sciences, University of Waterloo, Waterloo, Canada

**Keywords:** Fast food, Advertising to youth, Food environment, Marketing, Intake, Preferences

## Abstract

**Background:**

Consumption of fast food, which is associated with poor diet, weight gain and the development of noncommunicable diseases, is high amongst youth. Fast food marketing, a modifiable determinant of excess weight and obesity, affects youth’s food-related behaviours. This study aimed to examine the relationship between exposure to fast food marketing and the fast food brand preferences and intake amongst youth aged 10–17 across six countries.

**Methods:**

Data from 9,695 youth respondents living in Australia, Canada, Chile, Mexico, the United Kingdom (UK) and the United States (US) were analyzed from the 2019 International Food Policy Study (IFPS) Youth Survey. Survey measures assessed exposure to fast food marketing and brand-specific marketing, and preference for these brands and fast food intake. Regression models adjusted for age, sex, income adequacy and ethnicity were used to examine the associations.

**Results:**

Exposure to fast food marketing was positively associated with brand preferences and intake consistently across most countries. Overall, preference for McDonald’s (OR:1.97; 95% CI:1.52, 2.56), KFC (OR:1.61; 95% CI:1.24, 2.09) and Subway (OR:1.73; 95% CI:1.34, 2.24) were highest when exposed to general fast food marketing ≥ 2x/week compared to never. Preference for McDonald’s (OR:2.32; 95% CI:1.92, 2.79), KFC (OR:2.28; 95% CI:1.95, 2.68) and Subway (OR:2.75; 95% CI:2.32, 3.27) were also higher when exposed to marketing for each brand compared to not. Fast food intake was highest in Chile (IRR:1.90; 95% CI:1.45, 2.48), the UK (IRR:1.40; 95% CI:1.20, 1.63), Canada (IRR:1.32; 95% CI:1.19, 1.48), Mexico (IRR:1.26; 95% CI:1.05, 1.53) and the US (IRR:1.21; 95% CI:1.05, 1.41) when exposed to general fast food marketing ≥ 2x/week compared to never and was higher across most countries when exposed to brand-specific marketing compared to not. Respondents classified as ethnic minorities were more likely to report consuming fast food than ethnic majorities, and females were less likely to report consuming fast food than males.

**Conclusions:**

Exposure to fast food marketing is consistently and positively associated with brand preferences and fast food intake in all six countries. Our results highlight the need for strict government regulation to reduce exposure of unhealthy food marketing to youth in all six countries.

## Introduction

The burden caused by noncommunicable diseases (NCDs), such as cardiovascular disease, cancer and diabetes, is on the rise globally. In 2019, 20% of adolescent deaths worldwide occurred as a result of NCDs and it has been estimated that 70% of premature deaths in adults are linked to behaviours that developed during childhood and adolescence [1]. Diet, physical activity and lifestyle factors are modifiable precursors to obesity and excess weight that are an ongoing threat to health and the development of NCDs internationally [2]. Between 1975 and 2016, the prevalence of obesity and overweight amongst children and adolescents between the ages of 5 and 19 worldwide increased from 4 to 18%, alongside the intake of ultra-processed foods, high in sugar, saturated fats and sodium amongst youth [3, 4]. In Canada, youth aged 2–18 years consume over 50% of their total daily energy from ultra-processed food, elevating short- and long-term risks to health, including excess weight and obesity, mortality, and the development of noncommunicable diseases [5, 6].

Fast food accounts for a large share of food consumed by youth as on average, over 15% of daily calories consumed by North American youth come from such foods [7, 8]. Due to the poor nutrient quality of fast food, intake of these foods is associated with poor dietary quality and weight gain, and may compromise nutrient requirements necessary for proper growth [9, 10].

The food environment has been recognized as a determinant of obesity and the marketing of unhealthy foods and beverages to children has been identified as a cause of poor diet and excess weight in youth [11–13]. Youth are valuable advertising targets for the food and beverage industry, as promoting sales in this highly impressionable age group may help to create life-long brand loyalty [14–17]. Youth are exposed to food and beverage marketing (herein referred to as food marketing) daily in a variety of media and settings, which have the power to influence consumption and future health outcomes [10–13, 18–25]. Research from high-income countries found that the majority of advertisements on youth-oriented media promote unhealthy products and fast food in particular accounts for the largest exposure [19, 23, 26–32]. Expenditure data also shows that expenditures on youth-oriented advertising across all media is high and overall, the majority of advertising spend is devoted to unhealthy products, with fast food advertising dominating expenditures [22, 33]. This emphasis on fast food marketing is notable as youth spend a lot of time viewing various media and hold autonomous buying power [14–17]. In response to the ongoing concern caused by industry marketing practices and its negative impacts on youth health, in 2010, the World Health Organization recommended that its members develop restrictions to limit the marketing of foods high in fats, sugars and sodium (HFSS) to children [34]. Globally, food marketing restrictions are either non-existent, self-regulated by the food and beverage and/or advertising industries (e.g., Canada [excluding Quebec], Australia and the United States [35–38]) or government regulated (e.g., United Kingdom, Chile and Mexico [39–41]).

The logic model of unhealthy food promotion effects predicts that preferences and consumption of unhealthy foods are direct effects of food marketing exposure that eventually lead to long-term post consumption effects such as weight gain and diet-related disease, warranting investigation into its influence on youth [42]. Currently, research evaluating the impact of unhealthy food marketing on preferences and intake of youth globally is limited, as the few studies identified do not investigate more than one country, are focused on exposure from a specific media channel (mostly television), use a wide variety of data collection methods, rely on data collected from parents, and/or have a narrow age range and small sample size [43–55].

No previous studies have tested the association between youth’s self-reported exposure to and preference for specific fast food brands, nor does any investigate fast food marketing exposure, fast food restaurant brand preferences and fast food intake in this population simultaneously. Given that fast food is the most marketed food category to youth across most media [19, 22, 26, 30, 33], further investigation of its effects on youth is warranted. The purpose of this study was to examine the relationship between exposure to fast food marketing and the fast food brand preferences and intake of youth in six upper and middle income countries and to explore the relationship between sociodemographic characteristics and fast food preferences and intake.

## Methods

Data were from the 2019 International Food Policy Study (IFPS) Youth Survey, an annual repeat cross-sectional survey conducted in six countries: Australia, Canada, Chile, Mexico, UK and the US. Data were collected via self-completed web-based surveys conducted in November–December 2019 with youth aged 10–17 years. Respondents were recruited through parents/guardians enrolled in the Nielsen Consumer Insights Global Panel and their partners’ panels and invitation links were sent to panelists within each country. Those who confirmed they had a child aged 10–17 living in their household were asked for permission for their child to complete the survey, with quotas for age and sex groups in the UK and US. After eligibility screening, all potential respondents were provided with information about the study and asked to provide assent. Surveys were conducted in English in Australia and the UK; Spanish in Chile and Mexico; English or French in Canada; and English or Spanish in the US. Members of the research team who were native speakers in each language reviewed the French and Spanish translations independently. Brand marketing exposure and preference were assessed for McDonalds, KFC and Subway as these brands are among the global leaders in fast food service and have chains in each of the 6 countries [56]. The median survey time was 24 min [57].

The child’s parent/guardian received remuneration in accordance with their panel’s usual incentive structure (e.g., points-based or monetary rewards, etc.). A full description of the study methods can be found elsewhere [57].

### Measures

#### Independent Measures: Self-reported exposure to fast food marketing

Self-reported exposure to fast food marketing was assessed using two measures: general exposure to all instances of fast food marketing and exposure to only brand-specific fast food marketing. First, general exposure to fast food marketing was assessed using the following measure: *“In the last 30 days, how often did you see or hear advertisements for these kinds of food or drinks? Ads for fast food from a restaurant”.* The 6-item Likert scale for general exposure to fast food marketing was recategorized into the following: *“never” (“never”), “* ≤ *1x/week” (“less than once a week”, “once a week”),* and *“* ≥ *2x/week” (“a few times a week”, “every day”, “more than once a day”).* Second, self-reported exposure to McDonald’s, KFC and Subway marketing specifically, was assessed using the corresponding brand’s logo displayed with the following measure: “*Have you seen an advertisement for this restaurant in the last 30 days?”* (*“yes”, “no”, “don’t know”* or *“refuse to answer”*)*.*

#### Outcome Measures: Self-reported fast-food intake and fast food brand preference

**Fig. 1:**
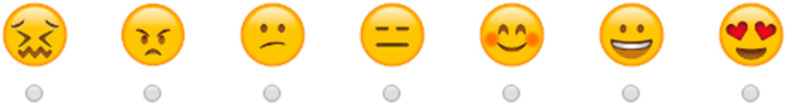
7-item emoji-based Likert scale used for the measurement of fast food brand preference

Self-reported intake of fast food was assessed using the following measure: “*Think about the last 7 days. How many days did you have a meal (breakfast, lunch or dinner) from restaurants, fast food places, food stands, or vending machines? (Don’t include meals at schools).”* Respondents had the option of selecting: a total number of days between 0–7, *“don’t know”* or *“refuse to answer”.* Self-reported preference for McDonald’s, KFC and Subway specifically, was assessed using the corresponding brand’s logo displayed with the following measure: “*How much would you like to go to this restaurant?”.* Respondents had the option of selecting from a 7-item emoji-scale, as displayed in Fig. 1. *“Don’t know”* and *“refuse to answer”* were also response options. The emoji-scale was recategorized into the following: *“not preferred”* (  ,  ,  ), *“neutral”* (  ), *“preferred”* (  ,  ,  ). For this measure, the sample was randomized to provide a response for only one of the three brands.

#### Sociodemographic measures

The sociodemographic measures included in this study were the respondent’s age, sex at birth, perceived income adequacy and ethnicity. Age was collected as a continuous variable. Sex at birth was collected as either *“male”* or *“female”.* Income adequacy was collected using the following measure: “*Does your family have enough money to pay for things your family needs?” (“not enough money”, “barely enough money”, “enough money”, “more than enough money”, “don’t know”* or *“refuse to answer”).* Perceived income adequacy was recategorized into a binary variable for either *“enough money”* (*“enough money”* and *“more than enough money”*) or *“not enough money”* (*“not enough money”* and *“barely enough money”*). Ethnicity was assessed using census measures from each country and re-coded to either “majority” or “minority” to derive comparable measures across countries.

#### Data analysis

The analytic sample included 11,108 respondents. A sub-sample of 9,695 respondents were included in the current analysis after excluding those with missing and/or incomplete data (i.e., *“don’t know”, “refuse to answer”* or left their answer selection blank) on sociodemographic characteristics, predictor variables and outcome variables (1,413 respondents; 12.7%). Sensitivity analyses indicate that excluded respondents were not different demographically to the final analytical sample. Data were weighted with post-stratification sample weights constructed using a raking algorithm with population estimates from the census in each country based on age group, sex, region in all countries, and ethnicity (except in Canada, where ethnicity wasn’t considered in the sample weights). All estimates reported throughout are weighted. Statistical analyses were conducted using SAS Studio OnDemand for Academics (SAS Institute Inc., 2021).

Ordinal logistic or negative binomial regression models were used to model the associations as appropriate. Each model was adjusted for age, sex, perceived income adequacy and ethnicity. Statistical significance for all models was set at an alpha level < 0.05, and significance was determined using a *p*-value < 0.05 or a 95% confidence interval. Two-way interaction terms were tested between country and each of the sociodemographic variables. Significant interactions were noted for the associations between youth’s self-reported general exposure to fast food brand-specific marketing and self-reported fast food intake (p < 0.05), and the association between youth’s self-reported exposure to brand-specific marketing and self-reported fast food intake (p < 0.05). Since some significant interactions were found, all results were stratified by country.

## Results

Weighted sample characteristics of youth respondents aged 10–17 in all six countries are presented in Table 1. Proportional differences in sociodemographic characteristics were noted across all countries. Overall, there was a higher proportion of adolescents aged 13–17 in all countries, the US had a higher proportion of minority respondents than other countries, and Canada had a higher proportion of respondents who perceived their family to have enough money compared to the other countries. In terms of general exposure to all fast food marketing, between 58–75% of respondents reported exposure ≥ 2x/week, with the greatest exposure reported in Mexico (75.3% of respondents) and the least exposure reported in the UK (58.7%), whereas between 17–26% of respondents reported exposure ≤ 1x/week with the greatest exposure reported in the UK (26.4%) and the least exposure reported in the US (17.3%).

**Table 1:**
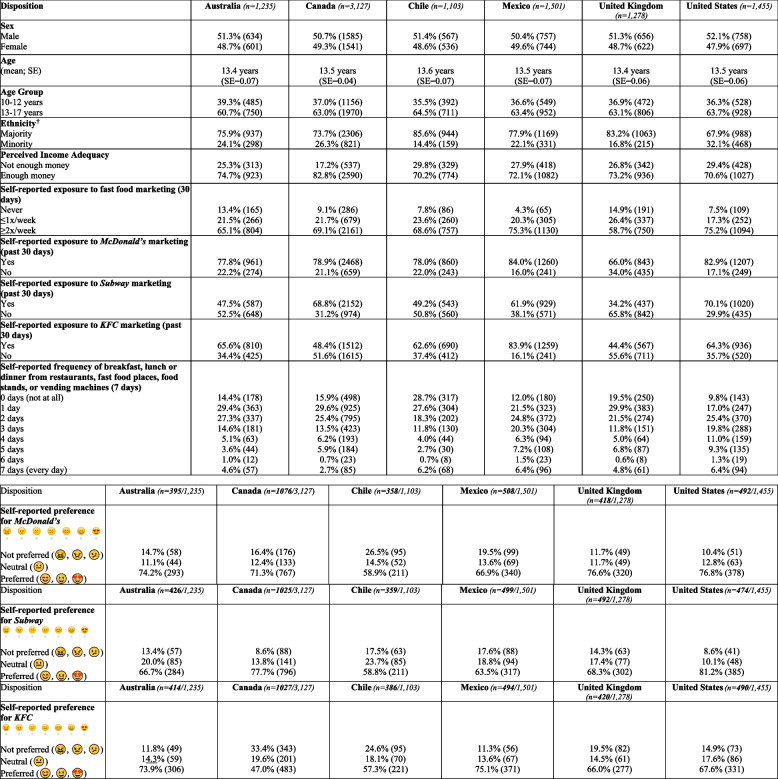
Sample characteristics of youth aged 10–17 in six countries (weighted) *N* = 9,695

^†^Ethnicity categories as per census questions asked in each country: 1) Canada majority = White, minority = other ethnicity; 2) Australia majority = only speaks English at home, minority = speaks a language besides English at home; 3) UK majority = White, minority = other ethnicity; 4) US majority = White, minority = other ethnicity; 5) Mexico majority = Non-indigenous, minority = indigenous; 6) Chile majority = Non-indigenous, minority = indigenous

### Association between youth’s self-reported general exposure to all fast food marketing and fast food brand preference

#### General exposure to all fast food marketing and preference for McDonald’s

Overall, the odds of preferring McDonald’s were significantly higher in the UK and the US and significantly lower in Mexico and Chile compared to Canada (Table 2). In terms of general exposure to fast food marketing, overall, respondents reportedly preferred McDonald’s most when exposed to general fast food marketing ≥ 2x/week (*OR:*1.97; *95% CI:* 1.52, 2.56) and ≤ 1x/week *(OR:*1.57; *95% CI:* 1.17, 2.10) compared to never being exposed to this marketing. Additionally, the odds of preferring McDonald’s decreased with increasing age.

**Table 2:** Overall odds ratio estimates from separate proportional odds regression models examining the association between general exposure to fast food marketing and preference for McDonald’s, KFC and Subway among youth in six countries

	**McDonald’s**		**KFC**		**Subway**	
**Parameter**	***OR***	***(95% CI)***	***OR***	***(95% CI)***	***OR***	***(95% CI)***
**Country**						
Canada	[ref]	[ref]	[ref]	[ref]	[ref]	[ref]
Chile	0.54	(0.42, 0.71)^a^	1.51	(1.19, 1.93)^a^	0.42	(0.32, 0.55)^a^
Mexico	0.75	(0.58, 0.99)^a^	3.49	(2.63, 4.63)^a^	0.48	(0.37, 0.62)^a^
United States	1.33	(1.01, 1.73)^a^	2.41	(1.89, 1.93)^a^	1.20	(0.90, 1.61)
United Kingdom	1.38	(1.04, 1.83)^a^	2.22	(1.72, 2.87)^a^	0.64	(0.49, 0.83)^a^
Australia	1.14	(0.87, 1.51)	3.39	(2.61, 4.39)^a^	0.60	(0.46, 0.77)^a^
**Age**	0.89	(0.85, 0.92)^a^	0.94	(0.91, 0.97)^a^	1.02	(0.99, 1.06)
**Sex**						
Male	[ref]	[ref]	[ref]	[ref]	[ref]	[ref]
Female	0.95	(0.80, 1.12)	0.72	(0.62, 0.84)^a^	1.06	(0.90, 1.25)
**Ethnicity**						
Majority	[ref]	[ref]	[ref]	[ref]	[ref]	[ref]
Minority	0.98	(0.79, 1.22)	1.16	(0.95, 1.43)	0.98	(0.79, 1.22)
**Income Adequacy**						
Not enough money	[ref]	[ref]	[ref]	[ref]	[ref]	[ref]
Enough money	0.82	(0.67, 1.01)	1.02	(0.85, 1.23)	1.11	(0.92, 1.35)
**General Exposure to FF Marketing**						
Never	[ref]	[ref]	[ref]	[ref]	[ref]	[ref]
≥ 2x/week	1.97	(1.52, 2.56)^a^	1.61	(1.24, 2.09)^a^	1.73	(1.34, 2.24)^a^
≤ 1x/week	1.57	(1.17, 2.10)^a^	1.54	(1.15, 2.07)^a^	1.46	(1.09, 1.97)^a^

*OR* Odds ratio, *CI* Confidence interval, *ref* Reference, *FF* Fast food

^a^Indicates significant test at an alpha level of 0.05

By country, the odds of preferring McDonald’s when exposed to general fast food marketing ≥ 2x/week compared to never being exposed in a week were greatest in the US, followed by the UK, Canada and Australia (Table 3).

**Table 3:** Odds ratio estimates from separate proportional odds regression models stratified by country examining the association between general exposure to fast food marketing and preference for McDonald’s, KFC and Subway among youth in six countries

	**Canada**		**Australia**		**United Kingdom**		**United States**		**Mexico**		**Chile**	
**Parameter**	***OR***	***(95% CI)***	***OR***	***(95% CI)***	***OR***	***(95% CI)***	***OR***	***(95% CI)***	***OR***	***(95% CI)***	***OR***	***(95% CI)***
***General Exposure to Fast Food Marketing and Preference for McDonald’s***												
**Age**	0.87	(0.82, 0.93)^a^	0.89	(0.80, 0.99)^a^	0.90	(0.80, 1.02)	0.88	(0.79, 0.98)^a^	0.92	(0.83, 1.11)	0.89	(0.80, 0.98)^a^
**Sex**												
Male	[ref]	[ref]	[ref]	[ref]	[ref]	[ref]	[ref]	[ref]	[ref]	[ref]	[ref]	[ref]
Female	0.91	(0.69, 1.20)	0.65	(0.39, 1.07)	0.97	(0.59, 1.59)	1.06	(0.67, 1.68)	1.19	(0.75, 1.88)	0.86	(0.55, 1.34)
**Ethnicity**												
Majority	[ref]	[ref]	[ref]	[ref]	[ref]	[ref]	[ref]	[ref]	[ref]	[ref]	[ref]	[ref]
Minority	0.83	(0.60, 1.13)	1.08	(0.57, 2.06)	0.63	(0.29, 1.36)	1.35	(0.84, 2.19)	1.12	(0.58, 2.17)	1.22	(0.62, 2.40)
**Income Adequacy**												
Not enough money	[ref]	[ref]	[ref]	[ref]	[ref]	[ref]	[ref]	[ref]	[ref]	[ref]	[ref]	[ref]
Enough money	0.84	(0.57, 1.26)	0.84	(0.49, 1.44)	0.94	(0.54, 1.66)	0.68	(0.40, 1.16)	1.01	(0.62, 1.66)	0.67	(0.40, 1.11)
**General Exposure to FF Marketing**												
Never	[ref]	[ref]	[ref]	[ref]	[ref]	[ref]	[ref]	[ref]	[ref]	[ref]	[ref]	[ref]
≥ 2x/week	2.02	(1.30, 3.15)^a^	2.02	(1.06, 3.86)^a^	2.20	(1.16, 4.18)^a^	2.28	(1.10, 4.71)^a^	1.24	(0.39, 3.89)	1.68	(0.72, 3.89)
≤ 1x/week	1.39	(0.85, 2.30)	1.19	(0.54, 2.60)	1.50	(0.75, 3.00)	2.78	(1.17, 6.64)^a^	1.22	(0.37, 4.04)	1.48	(0.60, 3.66)
***General Exposure to Fast Food Marketing and Preference for KFC***												
**Age**	0.89	(0.84, 0.94)^a^	0.99	(0.90, 1.10)	0.98	(0.87, 1.09)	1.01	(0.92, 1.11)	0.99	(0.88, 1.11)	0.93	(0.85, 1.02)
**Sex**												
Male	[ref]	[ref]	[ref]	[ref]	[ref]	[ref]	[ref]	[ref]	[ref]	[ref]	[ref]	[ref]
Female	0.55	(0.44, 0.71)^a^	0.51	(0.32, 0.83)^a^	0.95	(0.61, 1.49)	0.95	(0.62, 1.45)	0.91	(0.55, 1.51)	0.88	(0.57, 1.35)
**Ethnicity**												
Majority	[ref]	[ref]	[ref]	[ref]	[ref]	[ref]	[ref]	[ref]	[ref]	[ref]	[ref]	[ref]
Minority	1.37	(1.03, 1.83)^a^	0.96	(0.50, 1.85)	1.08	(0.52, 2.26)	1.23	(0.79, 1.89)	0.61	(0.31, 1.19)	1.51	(0.76, 3.02)
**Income Adequacy**												
Not enough money	[ref]	[ref]	[ref]	[ref]	[ref]	[ref]	[ref]	[ref]	[ref]	[ref]	[ref]	[ref]
Enough money	0.78	(0.57, 1.07)	0.90	(0.52, 1.55)	0.97	(0.60, 1.56)	1.44	(0.91, 2.30)	1.47	(0.85, 2.54)	1.13	(0.71, 1.79)
**General Exposure to FF Marketing**												
Never	[ref]	[ref]	[ref]	[ref]	[ref]	[ref]	[ref]	[ref]	[ref]	[ref]	[ref]	[ref]
≥ 2x/week	1.33	(0.88, 2.01)	2.15	(1.13, 4.06)^a^	1.99	(1.04, 3.78)^a^	0.95	(0.42, 2.14)	1.53	(0.52, 4.56)	2.34	(1.01, 5.44)^a^
≤ 1x/week	1.38	(0.87, 2.19)	2.81	(1.23, 6.41)^a^	1.60	(0.80, 3.21)	0.94	(0.37, 2.39)	1.33	(0.41, 4.36)	1.69	(0.67, 4.26)
***General Exposure to Fast Food Marketing and Preference for Subway***												
**Age**	0.99	(0.92, 1.06)	1.10	(1.00, 1.22)	0.99	(0.90, 1.09)	1.04	(0.93, 1.17)	1.05	(0.95, 1.15)	1.03	(0.93, 1.13)
**Sex**												
Male	[ref]	[ref]	[ref]	[ref]	[ref]	[ref]	[ref]	[ref]	[ref]	[ref]	[ref]	[ref]
Female	0.95	(0.70, 1.29)	1.12	(0.73, 1.74)	1.57	(1.02, 2.41)^a^	0.96	(0.58, 1.58)	1.03	(0.68, 1.56)	1.01	(0.64, 1.58)
**Ethnicity**												
Majority	[ref]	[ref]	[ref]	[ref]	[ref]	[ref]	[ref]	[ref]	[ref]	[ref]	[ref]	[ref]
Minority	0.90	(0.64, 1.27)	0.97	(0.54, 1.72)	1.95	(0.92, 4.15)	0.78	(0.46, 1.30)	0.98	(0.52, 1.84)	0.97	(0.47, 1.99)
**Income Adequacy**												
Not enough money	[ref]	[ref]	[ref]	[ref]	[ref]	[ref]	[ref]	[ref]	[ref]	[ref]	[ref]	[ref]
Enough money	1.01	(0.69, 1.49)	0.88	(0.53, 1.47)	0.79	(0.48, 1.31)	0.99	(0.58, 1.71)	1.38	(0.86, 2.22)	1.93	(1.20, 3.11)^a^
**General Exposure to FF Marketing**												
Never	[ref]	[ref]	[ref]	[ref]	[ref]	[ref]	[ref]	[ref]	[ref]	[ref]	[ref]	[ref]
≥ 2x/week	1.25	(0.75, 2.07)	1.84	(0.97, 3.49)	1.99	(1.10, 3.61)^a^	1.88	(0.77, 4.58)	2.80	(1.33, 5.91)^a^	1.57	(0.74, 3.33)
≤ 1x/week	1.10	(0.62, 1.96)	1.23	(0.60, 2.50)	1.43	(0.74, 2.79)	1.32	(0.45, 3.81)	2.48	(1.06, 5.82)^a^	2.02	(0.88, 4.64)

*OR* Odds ratio, *CI* Confidence interval, *ref* Reference, *FF* Fast food

^a^Indicates significant test at an alpha level of 0.05

#### General exposure to all fast food marketing and preference for KFC

Compared to Canada, overall, respondents from all countries were more likely to prefer KFC more, with the odds being highest in Mexico, followed by Australia, the US, the UK, and Chile (Table 2). Females were also less likely to prefer KFC than males by a factor of 0.72 (*95% CI:* 0.62, 0.84). In terms of general exposure to fast food marketing, the likelihood of preferring KFC was highest when respondents reportedly viewed this type of marketing ≥ 2x/week (*OR:*1.61; *95% CI:*1.24, 2.09) and ≤ 1x/week (*OR:*1.54; *95% CI:*1.15, 2.07) compared to not at all.

By country, the odds of preferring KFC when exposed to general fast food marketing ≥ 2x/week compared to not being exposed to this marketing at all were highest in Chile, followed by Australia and the UK (Table 3). In terms of sociodemographic characteristics, female respondents in Australia and Canada had a significantly lower preference for KFC compared to males, and in Canada, individuals who identified as a minority ethnicity preferred KFC significantly more than those who identified as a majority ethnicity.

#### General exposure to all fast food marketing and preference for Subway

Overall, compared to Canada, the likelihood of preferring Subway was significantly lower in most countries, with the lowest odds in Chile, followed by Mexico, Australia and the UK (Table 2). When respondents were exposed to general fast food marketing, the odds of preferring Subway was highest when exposed ≥ 2x/week (*OR:*1.73; *95% CI:*1.34, 2.24) and ≤ 1x/week (*OR:*1.46; *95% CI:*1.09, 1.97) compared to not being exposed at all.

By country, in Mexico and the UK, the odds of preferring Subway were 2.8 times (*95% CI:*1.33, 5.91) and 1.99 times greater (*95% CI:*1.10, 3.61), respectively, when exposed to general fast food marketing ≥ 2x/week compared to never being exposed to this marketing in a week (Table 3). With respect to sociodemographic characteristics, in the UK, females were 1.57 times more likely (*95% CI:*1.02, 2.41) to prefer Subway than males, and in Chile, those who reported perceiving their family to have enough money were 1.93 times more likely (*95% CI:*1.20, 3.11) to prefer Subway than those who perceived their family to not have enough money.

### Association between youth’s self-reported exposure to McDonald’s, Subway and KFC marketing and respective fast food brand preference

#### Exposure to only McDonald’s marketing and preference for McDonald’s

In all countries, more respondents reported being exposed to McDonald’s marketing than not (Table 1). Mexico had the greatest number of exposed respondents (84% of respondents), and the UK had the smallest number of exposed respondents (66%).

Similar to the models above, overall, the odds of preferring McDonald’s were significantly higher in the UK and the US and significantly lower in Chile and Mexico compared to Canada (Table 4). When exposed to McDonald’s marketing, the odds of respondents preferring McDonald’s were 2.32 times higher (*95% CI:*1.92, 2.79), compared to not being exposed. In terms of age, preference for McDonald’s decreased with increasing age.

**Table 4:** Overall odds ratio estimates from separate proportional odds regression models examining the association between exposure to McDonald’s, KFC and Subway marketing and preference for each respective brand among youth in six countries

	**McDonald’s Marketing Exposure and Preference for McDonald’s**		**KFC Marketing Exposure and Preference for KFC**		**Subway Marketing Exposure and Preference for Subway**	
**Parameter**	***OR***	***(95% CI)***	***OR***	***(95% CI)***	***OR***	***(95% CI)***
**Country**						
Canada	[ref]	[ref]	[ref]	[ref]	[ref]	[ref]
Chile	0.55	(0.42, 0.71)^a^	1.40	(1.09, 1.79)^a^	0.48	(0.36, 0.62)^a^
Mexico	0.76	(0.58, 0.99)^a^	2.71	(2.03, 3.62)^a^	0.53	(0.41, 0.69)^a^
United States	1.34	(1.02, 1.75)^a^	2.23	(1.74, 2.87)^a^	1.24	(0.93, 1.65)
United Kingdom	1.46	(1.10, 1.95)^a^	2.34	(1.81, 3.04)^a^	0.87	(0.67, 1.14)
Australia	1.09	(0.83, 1.44)	3.06	(2.36, 3.98)^a^	0.74	(0.57, 0.97)^a^
**Age**	0.89	(0.86, 0.93)^a^	0.94	(0.91, 0.97)	1.03	(0.99, 1.06)
**Sex**						
Male	[ref]	[ref]	[ref]	[ref]	[ref]	[ref]
Female	0.96	(0.81, 1.13)	0.73	(0.62, 0.85)^a^	1.03	(0.87, 1.21)
**Ethnicity**						
Majority	[ref]	[ref]	[ref]	[ref]	[ref]	[ref]
Minority	0.98	(0.79, 1.22)	1.13	(0.91, 1.39)	0.95	(0.77, 1.18)
**Income Adequacy**						
Not enough money	[ref]	[ref]	[ref]	[ref]	[ref]	[ref]
Enough money	0.84	(0.68, 1.03)	1.02	(0.84, 1.23)	1.09	(0.90, 1.32)
**Marketing Exposure to respective brand**						
Not exposed	[ref]	[ref]	[ref]	[ref]	[ref]	[ref]
Exposed	2.32	(1.92, 2.79)^a^	2.28	(1.95, 2.68)^a^	2.75	(2.32, 3.27)^a^

*OR* Odds ratio, *CI* Confidence interval, *ref* Reference

^a^Indicates significant test at an alpha level of 0.05

By country, the odds of preferring McDonald’s were greater when exposed to McDonald’s marketing as opposed to not being exposed, with the highest odds being in Chile, followed by Australia, Mexico, the US, Canada and the UK. (Table 5).

**Table 5:** Odds ratio estimates from separate proportional odds regression models stratified by country examining the association between exposure to McDonald’s, KFC and Subway marketing and preference for McDonald’s, KFC and Subway, respectively, among youth in six countries

	**Canada**		**Australia**		**United Kingdom**		**United States**	**Mexico**		**Chile**		
**Parameter**	***OR***	***(95% CI)***	***OR***	***(95% CI)***	***OR***	***(95% CI)***	***OR***	***(95% CI)***	***OR***	***(95% CI)***	***OR***	***(95% CI)***
***Exposure to McDonald’s Marketing and Preference for McDonald’s***												
**Age**	0.88	(0.83, 0.93)^a^	0.89	(0.80, 1.00)	0.91	(0.81, 1.03)	0.88	(0.79, 0.99)^a^	0.92	(0.83, 1.03)	0.90	(0.82, 1.00)
**Sex**												
Male	[ref]	[ref]	[ref]	[ref]	[ref]	[ref]	[ref]	[ref]	[ref]	[ref]	[ref]	[ref]
Female	0.95	(0.72, 1.25)	0.69	(0.42, 1.12)	0.95	(0.58, 1.54)	1.13	(0.71, 1.80)	1.19	(0.75, 1.89)	0.81	(0.52, 1.28)
**Ethnicity**												
Majority	[ref]	[ref]	[ref]	[ref]	[ref]	[ref]	[ref]	[ref]	[ref]	[ref]	[ref]	[ref]
Minority	0.85	(0.62, 1.18)	1.03	(0.54, 1.99)	0.62	(0.29, 1.34)	1.38	(0.85, 2.25)	1.02	(0.53, 1.97)	1.26	(0.66, 2.41)
**Income Adequacy**												
Not enough money	[ref]	[ref]	[ref]	[ref]	[ref]	[ref]	[ref]	[ref]	[ref]	[ref]	[ref]	[ref]
Enough money	0.84	(0.57, 1.26)	0.86	(0.50, 1.48)	0.95	(0.54, 1.68)	0.70	(0.41, 1.20)	1.06	(0.64, 1.74)	0.64	(0.38, 1.08)
**McDonald’s Marketing Exposure**												
Not Exposed	[ref]	[ref]	[ref]	[ref]	[ref]	[ref]	[ref]	[ref]	[ref]	[ref]	[ref]	[ref]
Exposed	2.08	(1.52, 2.85)^a^	2.87	(1.66, 4.95)^a^	2.08	(1.27, 3.40)^a^	2.12	(1.27, 3.53)^a^	2.28	(1.33, 3.91)^a^	3.22	(1.87, 5.52)^a^
***Exposure to KFC Marketing and Preference for KFC***												
**Age**	0.88	(0.83, 0.93)^a^	0.97	(0.88, 1.08)	0.97	(0.87, 1.08)	1.01	(0.92, 1.11)	1.00	(0.90, 1.12)	0.93	(0.85, 1.03)
**Sex**												
Male	[ref]	[ref]	[ref]	[ref]	[ref]	[ref]	[ref]	[ref]	[ref]	[ref]	[ref]	[ref]
Female	0.57	(0.44, 0.72)^a^	0.48	(0.30, 0.77)^a^	0.99	(0.64, 1.56)	0.97	(0.63, 1.48)	0.87	(0.53, 1.45)	0.94	(0.61, 1.43)
**Ethnicity**												
Majority	[ref]	[ref]	[ref]	[ref]	[ref]	[ref]	[ref]	[ref]	[ref]	[ref]	[ref]	[ref]
Minority	1.32	(0.99, 1.78)	0.94	(0.49, 1.77)	1.05	(0.49, 2.22)	1.22	(0.78, 1.89)	0.59	(0.30, 1.16)	1.44	(0.73, 2.89)
**Income Adequacy**												
Not enough money	[ref]	[ref]	[ref]	[ref]	[ref]	[ref]	[ref]	[ref]	[ref]	[ref]	[ref]	[ref]
Enough money	0.73	(0.53, 1.00)	0.96	(0.56, 1.63)	0.96	(0.59, 1.57)	1.35	(0.85, 2.16)	1.43	(0.83, 2.48)	1.19	(0.75, 1.90)
**KFC Marketing Exposure**												
Not Exposed	[ref]	[ref]	[ref]	[ref]	[ref]	[ref]	[ref]	[ref]	[ref]	[ref]	[ref]	[ref]
Exposed	2.80	(2.18, 3.59)^a^	2.59	(1.60, 4.20)^a^	2.35	(1.46, 3.79)^a^	1.87	(1.20, 2.90)^a^	2.02	(1.09, 3.74)^a^	1.64	(1.07, 2.52)^a^
***Exposure to Subway Marketing and Preference for Subway***												
**Age**	0.99	(0.93, 1.06)	1.11	(1.00, 1.22)	0.99	(0.90, 1.09)	0.99	(0.88, 1.11)	1.07	(0.97, 1.17)	1.01	(0.92, 1.12)
**Sex**												
Male	[ref]	[ref]	[ref]	[ref]	[ref]	[ref]	[ref]	[ref]	[ref]	[ref]	[ref]	[ref]
Female	0.95	(0.70, 1.29)	1.11	(0.72, 1.72)	1.53	(0.99, 2.37)	0.97	(0.59, 1.59)	0.89	(0.58, 1.35)	0.95	(0.61, 1.48)
**Ethnicity**												
Majority	[ref]	[ref]	[ref]	[ref]	[ref]	[ref]	[ref]	[ref]	[ref]	[ref]	[ref]	[ref]
Minority	0.88	(0.62, 1.24)	0.90	(0.51, 1.60)	1.95	(0.91, 4.19)	0.77	(0.46, 1.30)	0.83	(0.45, 1.53)	1.06	(0.53, 2.14)
**Income Adequacy**												
Not enough money	[ref]	[ref]	[ref]	[ref]	[ref]	[ref]	[ref]	[ref]	[ref]	[ref]	[ref]	[ref]
Enough money	1.03	(0.70, 1.52)	0.86	(0.52, 1.43)	0.78	(0.47, 1.30)	0.96	(0.56, 1.66)	1.34	(0.83, 2.15)	1.76	(1.08, 2.88)^a^
**Subway Marketing Exposure**												
Not Exposed	[ref]	[ref]	[ref]	[ref]	[ref]	[ref]	[ref]	[ref]	[ref]	[ref]	[ref]	[ref]
Exposed	2.26	(1.65, 3.10)^a^	2.64	(1.67, 4.18)^a^	3.44	(2.07, 5.72)^a^	4.20	(2.49, 7.06)^a^	2.66	(1.75, 4.02)^a^	2.78	(1.76, 4.38)^a^

*OR* Odds ratio, *CI* Confidence interval, *ref* Reference

^a^Indicates significant test at an alpha level of 0.05

#### Exposure to only KFC marketing and preference for KFC

In most countries, more respondents reported being exposed to KFC marketing than not (Table 1). Mexico had the greatest number of exposed respondents (83.9% of respondents), and the UK had the smallest number of exposed respondents (44.4%). Both the UK and Canada had more respondents who reported not being exposed to KFC marketing than being exposed (55.6% and 51.6%, respectively).

Similar to the previous models, compared to Canada, the odds of preferring KFC were significantly higher in all countries, with the highest odds of preference being in Australia, followed by Mexico, the UK, the US and Chile (Table 4). In terms of sex, females were less likely to prefer KFC than males. When reportedly viewing KFC marketing compared to not, the odds of preferring KFC were higher by a factor of 2.28 (*95% CI:* 1.95, 2.68).

By country, the odds of preferring KFC was higher in all countries when exposed to KFC marketing compared to not being exposed, with the greatest odds of preference in Canada, followed by Australia, the UK, Mexico, the US and Chile (Table 5). Females reportedly preferred KFC significantly less than males in Australia and Canada.

#### Exposure to only Subway marketing and preference for Subway

In the US, Canada and Mexico, more respondents reported being exposed to Subway marketing than not (70.1%, 68.8% and 61.9%, respectively) (Table 1). In the UK, Australia and Chile, more respondents reported not being exposed to Subway marketing than being exposed (65.8%, 52.5% and 50.8%, respectively).

Overall, the odds of preferring Subway were significantly lower in Chile, Mexico and Australia compared to Canada (Table 4). Additionally, respondents who reported being exposed to Subway marketing were significantly more likely to prefer Subway compared to those who were not exposed to this marketing (*OR:*2.75; *95% CI:*2.32, 3.27).

By country, the odds of preferring Subway in all countries was greater when exposed to Subway marketing compared to not being exposed, with the highest odds in the US, followed by the UK, Chile, Mexico, Australia and Canada (Table 5). In Chile, those who perceived their families to have enough money were more likely to prefer Subway than those who did not.

### Association between youth’s self-reported general exposure to all fast food marketing and fast food intake

In most countries, the odds of fast food intake were highest when exposed to general fast food marketing ≥ 2x/week compared to reportedly never being exposed, with the highest odds being in Chile, followed by the UK, Canada, Mexico and the US (Table 6). In terms of sociodemographic variables, in four countries, the odds of reported intake were significantly lower for females than males. Additionally, in almost all countries, the odds of reported fast food intake were significantly higher for those who identified as a minority compared to those who identified as a majority.

**Table 6:** Wald chi-square and contrast estimates (incidence rate ratios) from separate negative binomial regression models stratified by country examining the association between exposure to general fast food marketing and fast food intake among youth in six countries

	**General exposure to fast food marketing and fast food intake**											
	**Canada**		**Australia**		**United Kingdom**		**United States**		**Mexico**		**Chile**	
**Parameter**	***Wald χ***^***2***^ * (p)*	***IRR*** *(95% CI)*	***Wald χ***^***2***^* (p)*	***IRR*** *(95% CI)*	***Wald χ***^***2***^* (p)*	***IRR*** *(95% CI)*	***Wald χ***^***2***^* (p)*	***IRR*** *(95% CI)*	***Wald χ***^***2***^* (p)*	***IRR*** *(95% CI)*	***Wald χ***^***2***^* (p)*	***IRR*** *(95% CI)*
**Intercept**	**2.85** *(0.0913)*		**1.56** *(0.2111)*		**0.10** *(0.7575)*		**0.2686** *(0.0410)*^a^		**0.01** *(0.9196)*		**11.64** *(0.0006)*^a^	
**Age**	**56.13** *(*< *0.0001)*^a^		**16.96** *(*< *0.0001)*^a^		**13.93** *(0.0002)*^a^		**0.0325** *(*< *0.0001)*^a^		**21.52** *(*< *0.0001)*^a^		**17.88** *(*< *0.0001)*^a^	
**Sex**												
Male	[ref]	[ref]	[ref]	[ref]	[ref]	[ref]	[ref]	[ref]	[ref]	[ref]	[ref]	[ref]
Female	**11.92** *(0.0006)*^a^	**0.90** *(0.85, 0.96)*^a^	**15.43** *(*< *0.0001)*^a^	**0.83** *(0.76, 0.91)*^a^	**18.85** *(*< *0.0001)*^a^	**0.81** *(0.73, 0.89)*^a^	**0.13** *(0.7200)*	**0.99** *(0.92, 1.06)*	**1.19** *(0.2762)*	**0.96** *(0.90, 1.03)*	**5.62** *(0.0178)*^a^	**0.86** *(0.76, 0.97)*^a^
**Ethnicity**												
Majority	[ref]	[ref]	[ref]	[ref]	[ref]	[ref]	[ref]	[ref]	[ref]	[ref]	[ref]	[ref]
Minority	**17.79** *(*< *0.0001)*^a^	**1.15** *(1.08, 1.22)*^a^	**5.89** *(0.0152)*^a^	**1.15** *(1.03, 1.29)*^a^	**10.30** *(0.0013)*^a^	**1.26** *(1.09, 1.45)*^a^	**5.32** *(0.0210)*^a^	**1.09** *(1.01, 1.17)*^a^	**20.39** *(*< *0.0001)*^a^	**1.23** *(1.13, 1.35)*^a^	**2.34** *(0.1264)*	**1.15** *(0.96, 1.37)*
**Income Adequacy**												
Not enough money	[ref]	[ref]	[ref]	[ref]	[ref]	[ref]	[ref]	[ref]	[ref]	[ref]	[ref]	[ref]
Enough money	**0.65** *(0.4214)*	**0.97** *(0.90, 1.05)*	**0.57** *(0.4506)*	**1.04** *(0.94, 1.15)*	**2.05** *(0.1525)*^a^	**0.93** *(0.83, 1.03)*	**10.58** *(0.0011)*^a^	**1.14** *(1.05, 1.23)*^a^	**44.46** *(*< *0.0001)*^a^	**1.31** *(1.21, 1.42)*^a^	**1.72** *(0.1897)*	**1.10** *(0.96, 1.25)*
**General Exposure to FF Marketing**												
Never	[ref]	[ref]	[ref]	[ref]	[ref]	[ref]	[ref]	[ref]	[ref]	[ref]	[ref]	[ref]
≥ 2x/week	**25.62** *(*< *0.0001)*^a^	**1.32** *(1.19, 1.48)*^a^	**0.31** *(0.5787)*	**0.97** *(0.84, 1.10)*	**19.13** *(*< *0.0001)*^a^	**1.40** *(1.20, 1.63)*^a^	**6.80** *(0.0091)*^a^	**1.21** *(1.05, 1.41)*^a^	**5.99** *(0.0144)*^a^	**1.26** *(1.05, 1.52)*^a^	**21.61** *(*< *0.0001)*^a^	**1.90** *(1.45, 2.48)*^a^
≤ 1x/week	**9.77** *(0.0018)*^a^	**1.21** *(1.07, 1.37)*^a^	**1.09** (0.2961)	**0.92** *(0.78, 1.08)*	**15.80** *(*< *0.0001)*^a^	**1.40** *(1.19, 1.65)*^a^	**1.48** *(0.2232)*	**1.11** *(0.94, 1.31)*	**0.87** *(0.3519)*	**1.10** *(0.90, 1.34)*	**12.96** *(0.0003)*^a^	**1.70** *(1.27, 2.27)*^a^

*IRR* Incidence rate ratio, *χ2* Chi-square, *CI* Confidence interval, *ref* Reference, *FF* Fast food

^a^Indicates significant test at an alpha level of 0.05

### Association between youth’s self-reported exposure to only McDonald’s, KFC or Subway marketing and fast food intake

#### Fast food intake and exposure to only McDonald’s marketing

In almost all countries, the odds of reported fast food intake were higher for those who were reportedly exposed to McDonald’s marketing compared to those who were not exposed, with the highest odds being in Chile, followed by Canada, the UK, the US and Mexico (Table 7). With respect to sociodemographic characteristics, in the UK, Australia, Canada and Chile, the odds of reportedly consuming fast food were significantly lower for females than males. With regard to ethnicity, in almost all countries, the odds of reportedly eating fast food was significantly higher amongst those who identified as a minority in their country as opposed to a majority.

**Table 7:** Wald chi-square and contrast estimates (incidence rate ratios) from separate negative binomial regression models stratified by country examining the association between exposure to McDonald’s, KFC and Subway marketing and fast food intake among youth in six countries

	**Canada**			**Australia**			**United Kingdom**		**United States**		**Mexico**		**Chile**	
**Parameter**	***Wald χ***^***2***^* (p)*	***IRR*** *(95% CI)*		***Wald χ***^***2***^* (p)*		***IRR*** *(95% CI)*	***Wald χ***^***2***^* (p)*	***IRR*** *(95% CI)*	***Wald χ***^***2***^* (p)*	***IRR*** *(95% CI)*	***Wald χ***^***2***^* (p)*	***IRR*** *(95% CI)*	***Wald χ***^***2***^* (p)*	***IRR*** *(95% CI)*
***McDonald’s marketing exposure and fast food intake***														
**Intercept**	**2.37** *(0.1239)*			**1.14** *(0.2851)*			**0.17** *(0.6772)*		**5.50** *(0.0190)*^a^		**0.37** *(0.5430)*		**7.39** *(0.0065)*^a^	
**Age**	**61.06** *(*< *0.0001)*^a^			**17.59** *(*< *0.0001)*^a^			**15.71** *(*< *0.0001)*^a^		**18.93** *(*< *0.0001)*^a^		**23.45** *(*< *0.0001)*^a^		**20.05** *(*< *0.0001)*^a^	
**Sex**														
Male	[ref]	[ref]		[ref]		[ref]	[ref]	[ref]	[ref]	[ref]	[ref]	[ref]	[ref]	[ref]
Female	**11.26** *(0.0008)*^a^	**0.91** *(0.86, 0.96)*^a^		**15.15** *(*< *0.0001)*^a^		**0.83** *(0.76, 0.91)*^a^	**18.88** *(*< *0.0001)*^a^	**0.81** *(0.73, 0.89)*^a^	**0.08** *(0.7774)*	**0.99** *(0.92, 1.06)*	**1.00** *(0.3184)*	**0.97** *(0.90, 1.03)*	**4.41** *(0.0357)*^a^	**0.88** *(0.78, 0.99)*^a^
**Ethnicity**														
Majority	[ref]	[ref]		[ref]		[ref]	[ref]	[ref]	[ref]	[ref]	[ref]	[ref]	[ref]	[ref]
Minority	**17.72** *(*< *0.0001)*^a^	**1.15** *(1.08, 1.22)*^a^		**5.78** *(0.0162)*^a^		**1.15** *(1.03, 1.29)*^a^	**10.43** *(0.0012)*^a^	**1.26** *(1.10, 1.45)*^a^	**5.36** *(0.0206)*^a^	**1.09** *(1.01, 1.17)*^a^	**21.04** *(*< *0.0001)*^a^	**1.24** *(1.13, 1.36)*^a^	**1.84** *(0.1747)*	**1.13** *(0.95, 1.35)*
**Income Adequacy**														
Not enough money	[ref]	[ref]		[ref]		[ref]	[ref]	[ref]	[ref]	[ref]	[ref]	[ref]	[ref]	[ref]
Enough money	**0.64** *(0.4240)*	**0.97** *(0.90, 1.05)*		**0.48** *(0.4905)*		**1.04** *(0.94, 1.15)*	**2.39** *(0.1224)*	**0.92** *(0.83, 1.02)*	**10.93** *(0.0009)*^a^	**1.14** *(1.05, 1.23)*^a^	**46.47** *(*< *0.0001)*^a^	**1.32** *(1.22, 1.43)*^a^	**1.88** *(0.1704)*	**1.10** *(0.96, 1.26)*
**McDonald’s Marketing Exposure**														
Not exposed	[ref]	[ref]		[ref]		[ref]	[ref]	[ref]	[ref]	[ref]	[ref]	[ref]	[ref]	[ref]
Exposed	**37.03** *(*< *0.0001)*^a^	**1.26** *(1.17, 1.35)*^a^		**0.09** *(0.7669)*		**0.98** *(0.88, 1.10)*	**14.84** *(0.0001)*^a^	**1.23** *(1.11, 1.36)*^a^	**9.11** *(0.0025)*^a^	**1.16** *(1.05, 1.28)*^a^	**7.43** *(0.0064)*^a^	**1.14** *(1.04, 1.26)*^a^	**19.52** *(*< *0.0001)*^a^	**1.43** *(1.22, 1.68)*^a^
***KFC marketing exposure and fast food intake***														
**Intercept**	**1.79** *(0.1815)*			**0.41** *(0.5196)*			**0.55** *(0.4595)*		**5.89** *(0.0152)*^a^		**0.70** *(0.4036)*		**4.11** *(0.0426)*^a^	
**Age**	**61.81** *(*< *0.0001)*^a^			**17.67** *(*< *0.0001)*^a^			**14.30** *(0.0002)*^a^		**19.44** *(*< *0.0001)*^a^		**22.94** *(*< *0.0001)*^a^		**19.52** *(*< *0.0001)*^a^	
**Sex**														
Male	[ref]		[ref]		[ref]		[ref]	[ref]	[ref]	[ref]	[ref]	[ref]	[ref]	[ref]
Female	**8.22** *(0.0041)*^a^		**0.92** *(0.87, 0.97)*^a^		**15.52** *(*< *0.0001)*^a^	**0.83** *(0.76, 0.91)*^a^	**17.79** *(*< *0.0001)*^a^	**0.81** *(0.74, 0.90)*^a^	**0.05** *(0.8151)*	**0.99** *(0.93, 1.06)*	**1.06** *(0.3036)*	**0.97** *(0.90, 1.03)*	**3.91** *(0.0480)*^a^	**0.88** *(0.78, 0.99)*^a^
**Ethnicity**														
Majority	[ref]		[ref]		[ref]	[ref]	[ref]	[ref]	[ref]	[ref]	[ref]	[ref]	[ref]	[ref]
Minority	**10.57** *(0.0012)*^a^		**1.11** *(1.04, 1.18)*^a^		**5.25** *(0.0220)*^a^	**1.14** *(1.02, 1.28)*^a^	**8.79** *(0.0030)*^a^	**1.24** *(1.07, 1.42)*^a^	**5.17** *(0.0229)*^a^	**1.09** *(1.01, 1.17)*^a^	**20.92** *(*< *0.0001)*^a^	**1.24** *(1.13, 1.35)*^a^	**1.80** *(0.1794)*	**1.13** *(0.95, 1.35)*
**Income Adequacy**														
Not enough money	[ref]		[ref]		[ref]	[ref]	[ref]	[ref]	[ref]	[ref]	[ref]	[ref]	[ref]	[ref]
Enough money	**0.44** *(0.5084)*		**0.98** *(0.91, 1.05)*		**0.66** *(0.4166)*	**1.04** *(0.94, 1.16)*	**2.60** *(0.1070)*	**0.92** *(0.82, 1.02)*	**9.12** *(0.0025)*^a^	**1.13** *(1.04, 1.22)*^a^	**46.07** *(*< *0.0001)*^a^	**1.32** *(1.22, 1.43)*^a^	**2.29** *(0.1303)*	**1.11** *(0.97, 1.27)*
**KFC Marketing Exposure**														
Not exposed	[ref]		[ref]		[ref]	[ref]	[ref]	[ref]	[ref]	[ref]	[ref]	[ref]	[ref]	[ref]
Exposed	**109.58** *(*< *0.0001)*^a^		**1.35** *(1.28, 1.43)*^a^		**2.51** *(0.1130)*	**1.08** *(0.98, 1.19)*	**26.84** *(*< *0.0001)*^a^	**1.29** *(1.17, 1.42)*^a^	**28.06** *(*< *0.0001)*^a^	**1.22** *(1.13, 1.31)*^a^	**4.89** *(0.0270)*^a^	**1.11** *(1.01, 1.23)*^a^	**8.88** *(0.0029)*^a^	**1.22** *(1.07, 1.39)*^a^
***Subway marketing exposure and fast food intake***														
**Intercept**	**0.22** *(0.6409)*				**0.48** *(0.4906)*		**0.54** *(0.4637)*		**0.54** *(0.4637)*		**0.76** *(0.3831)*		**0.76** *(0.3831)*	
**Age**	**59.94** *(*< *0.0001)*^a^				**17.63** *(*< *0.0001)*^a^		**15.84** *(*< *0.0001)*^a^		**15.84** *(*< *0.0001)*^a^		**21.28** *(*< *0.0001)*^a^		**21.28** *(*< *0.0001)*^a^	
**Sex**														
Male	[ref]		[ref]		[ref]	[ref]	[ref]	[ref]	[ref]	[ref]	[ref]	[ref]	[ref]	[ref]
Female	**10.91** *(0.0010)*^a^		**0.91** *(0.86, 0.96)*^a^		**15.20** *(*< *0.0001)*^a^	**0.83** *(0.76, 0.91)*^a^	**17.65** *(*< *0.0001)*^a^	**0.81** *(0.74, 0.90)*^a^	**17.65** *(*< *0.0001)*^a^	**0.98** *(0.92, 1.05)*	**1.79** *(0.1808)*	**0.96** *(0.89, 1.02)*	**1.79** *(0.1808)*	**0.86** *(0.76, 0.97)*^a^
**Ethnicity**														
Majority	[ref]		[ref]		[ref]	[ref]	[ref]	[ref]	[ref]	[ref]	[ref]	[ref]	[ref]	[ref]
Minority	**17.41** *(*< *0.0001)*^a^		**1.15** *(1.08, 1.22)*^a^		**5.01** *(0.0252)*^a^	**1.14** *(1.02, 1.28)*^a^	**9.68** *(0.0019)*^a^	**1.25** *(1.09, 1.44)*^a^	**9.68** *(0.0019)*^a^	**1.09** *(1.01, 1.17)*^a^	**18.80** *(*< *0.0001)*^a^	**1.22** *(1.12, 1.34)*^a^	**18.80** *(*< *0.0001)*^a^	**1.12** *(0.94, 1.34)*
**Income Adequacy**														
Not enough money	[ref]		[ref]		[ref]	[ref]	[ref]	[ref]	[ref]	[ref]	[ref]	[ref]	[ref]	[ref]
Enough money	**0.88** *(0.3495)*		**0.96** *(0.90, 1.04)*		**0.66** *(0.4182)*	**1.04** *(0.94, 1.16)*	**2.16** *(0.1418)*	**0.92** *(0.83, 1.03)*	**10.14** *(0.0015)*^a^	**1.13** *(1.05, 1.22)*^a^	**41.36** *(*< *0.0001)*^a^	**1.30** *(1.20, 1.41)*^a^	**41.36** *(*< *0.0001)*^a^	**1.08** *(0.95, 1.24)*
**Subway Marketing Exposure**														
Not exposed	[ref]		[ref]		[ref]	[ref]	[ref]	[ref]	[ref]	[ref]	[ref]	[ref]	[ref]	[ref]
Exposed	**13.12** *(0.0003)*^a^		**1.12** *(1.05, 1.20)*^a^		**4.32** *(0.0376)*^a^	**1.10** *(1.01, 1.20)*^a^	**20.25** *(*< *0.0001)*^a^	**1.25** *(1.14, 1.38)*^a^	**11.69** *(0.0006)*^a^	**1.14** *(1.06, 1.24)*^a^	**33.23** *(*< *0.0001)*^a^	**1.23** *(1.15, 1.32)*^a^	**33.23** *(*< *0.0001)*^a^	**1.33** *(1.17, 1.50)*^a^

*IRR* Incidence rate ratio, *χ2* Chi-square, *CI* Confidence interval, *ref* Reference

^a^Indicates significant test at an alpha level of 0.05

#### Fast food intake and exposure to only KFC marketing

In almost all countries, the odds of reportedly consuming fast food were higher for those who were reportedly exposed to KFC marketing compared to those who were not, with the highest odds being in Canada, followed by the UK, the US, Chile and Mexico (Table 7). In terms of sex, in four countries, females reportedly ate fast food significantly less than males. In almost all countries, the odds of consuming fast food were higher amongst those who identified as a minority compared to those who identified as a majority.

#### Fast food intake and exposure to only Subway marketing

In all countries, the odds of reportedly eating fast food was significantly higher when exposed to Subway marketing as opposed to not being exposed, with the highest odds being in Chile, followed by the UK, Mexico, the US, Canada and Australia (Table 7). In terms of sex, in four countries, females reportedly ate fast food significantly less than males. The odds of consuming fast food were also significantly higher for those who identified as a minority compared to those who identified as a majority in almost all countries.

## Discussion

Overall, positive associations were found between exposure to fast food marketing and fast food brand preferences and intake. Preference for specific fast food brands was generally highest across countries when exposed to general fast food marketing ≥ 2x/week and ≤ 1x/week compared to those who were not exposed, and also higher among those who self-reported exposure to marketing for each respective brand compared to those who did not, and this relationship was consistent across all countries. In terms of fast food intake, reported consumption was generally highest across countries when exposed to general fast food marketing ≥ 2x/week and ≤ 1x/week compared to those who were not exposed. Across almost all countries, reported consumption of fast food was higher amongst those who were exposed to marketing for McDonald’s, KFC and Subway as opposed to those who were not. With respect to sociodemographic characteristics, across most countries overall, respondents who identified as a minority ethnicity were more likely to consume fast food than those of a majority ethnicity, and females were less likely to reportedly consume fast food than males.

The study findings suggest that the likelihood of preferring a fast food brand and consuming fast food increased with both exposure to brand-specific and general fast food marketing. These findings are consistent with previous epidemiological evidence assessing the association between food marketing that is not food category specific and health behaviours including youth’s intake and preferences, and also consistent with similarly designed cross-sectional observational studies among adults and younger age groups and specific food categories [43, 58–65]. Our findings build on this current body of knowledge by providing evidence for these associations for fast food specifically, which is important since it is the most marketed of all food categories [19, 22, 26, 30, 33]. This study also found that the odds of preferring a brand were higher overall across models when variables included recall of brand-specific fast food marketing, as opposed to more general exposure to fast food marketing. This may indicate that fast food brand-specific marketing has a greater effect on youth’s preferences for the respective brand compared to general fast food marketing, which would be consistent with data from other fields of research investigating the association between cigarette brand-specific marketing and brand preferences amongst adolescents and young adults [66, 67]. This stronger association may also be due to improved recall of instances of brand-specific marketing (compared to general instances of fast food marketing), as well as the type of questions asked (e.g., brand-specific marketing exposure was measured using a response of *“yes”* or *“no”*, compared to general marketing exposure which was assessed using a 6-item Likert scale). To help address this, the 6-item scale was re-categorized into a 3-item scale, but the associations amongst the brand-specific measure remained stronger. Although the results were largely consistent across countries, we cannot fully conclude from this study alone that these associations are causal, due to the self-reported, cross-sectional nature of the data. For example, the association between marketing exposure and food intake could be bidirectional in nature: it is possible that greater intake of certain fast food brands may also lead to increased exposure/attention to brand-specific marketing. However, our results are supported by existing epidemiological data and will also help to strengthen existing evidence on associations between exposure to unhealthy food marketing and increased preference and consumption [68].

Overall, the country-stratified results were fairly consistent across countries. As mentioned previously, the policy environments restricting unhealthy food marketing to children differ in stringency across the countries investigated, but yet, exposures are still high and the relationships between these exposures and eating behaviours are consistently strong across countries. Although most existing policies apply to children under the age of 14 and this study investigated those 10–17 years old, these findings still indicate that fast food marketing exposure is affecting the eating behaviours of youth and that current regulatory policies need to be strengthened to raise age thresholds beyond children, adopt more specific and uniform definitions for what is considered child marketing and implement more stringent HFSS thresholds.

This comprehensive survey also allowed for exploration of sociodemographic differences within the measured associations. Overall, females in most countries were less likely to report consumption of fast food than males, which is congruent with previous research measuring fast food intake [69–71]. An explanation for this consistent finding could be that female youth are more likely to engage in diet-related practices and are more attentive to their body image [72, 73]. It may also be possible that males are targeted by industry marketing practices more often than females, as males are reportedly featured more frequently in food marketing, which could lead to greater persuasion towards consuming the product [74]. We also found that individuals classified as ethnic minorities were more likely to report the consumption of fast food than ethnic majorities. Recent data has suggested that Black and Hispanic youth in the US are being disproportionally exposed to more unhealthy food marketing, which brings concern as socioeconomic status is associated with ethnic minority status in countries like the US, and those with a lower socioeconomic status are more likely to exhibit poorer health outcomes [75–82]. Thus, the marketing unhealthy foods may be exacerbating poor health outcomes in already at-risk populations. Implementing stringent regulations to protect youth from exposure to unhealthy food marketing may help to reduce these differences [77].

## Strengths and limitations

To our knowledge, this is the first study to examine associations between specific fast food brand marketing exposure and youth-reported intake and preferences. This study employs consistent measures across a large sample size with a wide age range and includes respondents from a variety of ethnicities and socioeconomic backgrounds in six different countries, which allows for greater generalizability and between country comparisons. Post-stratification weights were also used to provide a more representative sample, which also increases the generalizability of our findings. Additionally, as the exposure measures did not specifically focus on marketing in particular types of media, this allowed us to report our associations based on a wide range of exposures.

Interpretation of the findings should consider potential limitations of self-reported data. In addition to being subject to recall bias and reverse causation, the self-reported exposure variables do not examine the power, ad content, frequency, and extent to which it targets the individual. Past research has shown that certain marketing techniques affect one’s recall of the advertisement, which could have altered their ability to remember marketing exposures [83]. While the self-reported fast food intake variable technically includes food intake from settings beyond fast food places (i.e., restaurants, food stands or vending machines), these other sources can arguably also be considered fast food-like, due to the ease of purchase and poor nutrient content of most foods sold from these sources. Additionally, it is possible that what respondents encompassed under ‘fast food advertising’ may have been interpreted differently by individuals, introducing additional bias. Aside from its limitations, self-reported measures are also valuable in that they are more feasible to collect. Objective measures are often more difficult to gather, as they are more resource-intense and do not necessarily accurately represent day-to-day choices [68]. Furthermore, existing evidence suggests that self-reported exposure measures are correlated with objective exposure measures [84, 85]. The increased feasibility of self-reported measures also allows for more frequent monitoring and the ability to collect and compare data across multiple countries simultaneously.

Additionally, recruitment was completed using nonprobability-based sampling, meaning these findings may not be representative of national estimates. However, data were weighted by age group, sex, region, and ethnicity (except in Canada), which should mitigate this even if it did not completely remove the effect.

This study did not analyze these data by marketing policy jurisdiction, due to the complexities and differences in the policy inclusions/exclusions across the 6 countries and the cross-sectional nature of the data that cannot adjust for secular trends, as well as the sample not including children under the age of 10.

## Conclusion

Overall, we found positive associations between exposure to fast food marketing and the brand preferences and reported intake of youth across all six countries. Regardless of the policy landscape surrounding restricting unhealthy food marketing to children, it is evident that exposure to fast food marketing is negatively influencing youth’s preference for and intake of these foods, as evidence has suggested that the odds of becoming overweight or developing obesity increases with fast food consumption [86]. The results demonstrate that current efforts to limit marketing to children and youth are not effective. As such, more comprehensive and stringent government regulation restricting fast food marketing to youth in all media may help reduce preferences and consumption of fast food. Including adolescents in these restrictions is also important, as they hold independent purchasing power, are easily influenced, spend a lot of time watching screens and have a high consumption of fast food products [24, 25, 71, 87]. Future research should examine if and how these modelled associations differ by child and adolescent age groups. This research could provide preliminary evidence on the likely influence of marketing exposure on older youth on whom there is little research [64] and to investigate whether existing policies protecting children under 13 years old are effective in reducing exposure to fast food marketing and its consequences, such as brand preferences and intake.

## Data Availability

The data that support the findings from this study are available from the corresponding author under reasonable request.

## Abbreviations

NCDs: Noncommunicable diseases
HFSS: High in fat, sugar and sodium
IFPS: International Food Policy Study
KFC: Kentucky Fried Chicken
OR: Odds ratio
CI: Confidence interval
ref: Reference
FF: Fast food
IRR: Incidence rate ratio
